# Medical News and Miscellany

**Published:** 1892-08

**Authors:** 


### Medical News and Miscellany.

What's in a Name?—Iodoform would smell as bad by any other name.

---

The "Doctor's Crop."—A valued correspondent writes: "We had a fine rain Saturday, which will help the Doctor's crop—the "top-crop" of cotton.

---

In view of the possibility of an invasion of yellow fever from Mexico—the disease being at Vera Cruz—State Health Officer Swearingen has doubled his guards at Laredo and Eagle Pass.

---

In our write up of the College faculty in the *souvenir* edition the biography of Prof. A. J. Smith did not appear, from the fact that the Doctor failed to furnish the necessary data, much to our regret.

---

**Stories of a Country Doctor**, by Dr. Willis P. King. The third edition of this great book is now ready. Price $1, in cloth. With DANIEL'S TEXAS MEDICAL JOURNAL, post paid, $2.65; this office.

---

**Dr. Wm. Caston**, formerly a prominent practitioner of Texas at Corsicana and other points, and still a member of the State Medical Association, has removed from Spokane Falls, Washington, where he has resided several years, to Denver, Colorado.

---

**Death of Dr. Hardy's Son.**—Eddie, son of Dr. L. H. Hardy, of Paige, Texas, died on 16th of July ult., after a long and painful illness. He was just past eighteen, and was a very bright and promising young man. The JOURNAL tenders its sincere sympathy to the doctor in his severe bereavement.

---

**Errata.**—In our July number there occurred several provoking errors; the name of Dr. R. C. Hodges was spelled Hodge; Dr. H. P. Cooke's name was spelled "Cook." In Dr. H. Atchison West's name the middle name was spelled "Atcheson" and his place of nativity, Russell's Cave, was printed "Rupell's Cave." The word spectator was printed "speculator," and "extended trip" was printed "intended trip," etc. "Excuse these tears."

---

**Dr. L. M. Cocke**, who for several years has practiced medicine in San Marcos and other points in Hays county, and was at one time at Laredo, is now engaged in the manufacture and sale of a fumigating apparatus,—Bozart's Weavil Destroyer,—which apparatus, the Doctor claims, will not only destroy weavils in corn in bulk but will dininfect anything. The JOURNAL wishes the Doctor as much success as his energy and pluck entitle him to, and that is abundant.

It is far from our intention to do any one injustice. In last issue it was stated that Dr. West, Secretary Texas State Medical Association, drew his entire salary in advance, to wit: July 28th. Our attention has been called to the fact that he had at that time about gotten the Transactions ready, and they were issued in August. Thus, a part of the work had been done, and a part of the money was earned. Still, the salary was drawn, as stated, within ninety days of his induction into office. The established precedent for years was to pay the Secretary at next meeting. This would have given him his pay at Tyler.

---

**Removals, etc.**—Dr. S. S. Boyer, late of Galveston—an old army surgeon—has located at Waco.

Dr. E. C. Dallas has removed from Forest to Alto, Texas.

Dr. J. S. Steel, who graduated at Vanderbilt Medical College last session, has finally located at Wimberly's Mills, Hays county, Texas.

Dr. F. A. Schmitt, so long resident at Schulenburg, Tex., has removed to LaGrange, Fayette county.

Prof. Allen J. Smith, of the Texas Medical College, is spending his vacation at York, Penn. Will return in time for the fall session, which begins October 3rd.

---

**Kind Words.**—Among the numerous letters of commendation and words of encouragement that have been showered upon the JOURNAL since the appearance of its souvenir edition (July, '92), none are more highly prized than the following, extracted from a letter from an old Mississippi friend and subscriber—an ex-President of the Mississippi State Medical Association—Dr. N. L. Guice, of Natchez:

"It is my pleasure to congratulate you upon your success in elevating the JOURNAL to the position of *one of the very best* published in the Southwest! I well remember your efforts in this State when, as a member of our State Association, you ever advocated and insisted upon a higher standard of professional character; in fact, in all things connected with medicine; and your success in the great State of Texas impresses me favorably with the profession there, indicating, as it does, their appreciation of your efforts and a preference for a high standard of ability and morals. May the JOURNAL continue to flourish," etc.

### DEATH OF MRS. BENNETT.

After an illness of six weeks the wife of Dr. T. J. Bennett breathed her last at 8 o'clock p. m., August 8th inst. Her maiden name was Eupha Amanda Hume, and she was the sole surviving daughter of Joseph L. and Josephine Hume, her sister, Mrs. Walter Swain, it will be remembered, having died September 13, 1889.

Mrs. Bennett was born in Illinois, February 11, 1860, and with her parents moved to Austin in 1874. She married Dr. T. J. Bennett, December 30, 1885. She was the first graduate of St. Mary's Academy. She united with the Cumberland Presbyterian church about ten years ago, and has lived a devoted Christian life. She was a noble woman, warm-hearted, kind and generous, and a general favorite wherever known, and the news of her untimely death will carry surprise and sorrow to many an Austin home. To the stricken husband, father and mother the kindliest sympathy of this entire community goes out. Her life, in its sweetness and devotion to them, was their joy and comfort; around her death gathers a sadness deeper than the silent quiet of the tomb.—*Statesman.*

The funeral services were held at the family residence and at the Cumberland Presbyterian church, and were most beautiful and impressive.

